# Exploring regional aspects of 3D facial variation within European individuals

**DOI:** 10.1038/s41598-023-30855-x

**Published:** 2023-03-06

**Authors:** Franziska Wilke, Noah Herrick, Harold Matthews, Hanne Hoskens, Sylvia Singh, John R. Shaffer, Seth M. Weinberg, Mark D. Shriver, Peter Claes, Susan Walsh

**Affiliations:** 1 Department of Biology, Indiana University-Purdue University Indianapolis, 723 W Michigan St, Indianapolis, IN 46202 USA; 2 Department of Human Genetics, KU Leuven, Leuven, Belgium; 3 Murdoch Children’s Research Institute, Melbourne, VIC Australia; 4 Department of Human Genetics, University of Pittsburgh, Pittsburgh, PA USA; 5 Department of Oral and Craniofacial Sciences, Center for Craniofacial and Dental Genetics, University of Pittsburgh, Pittsburgh, PA USA; 6 Department of Anthropology, University of Pittsburgh, Pittsburgh, PA USA; 7 Department of Anthropology, The Pennsylvania State University, University Park, PA USA; 8 Medical Imaging Research Center, University Hospitals Leuven, Leuven, Belgium; 9 Department of Electrical Engineering, ESAT/PSI, KU Leuven, Leuven, Belgium

**Keywords:** Population genetics, Biological anthropology

## Abstract

Facial ancestry can be described as variation that exists in facial features that are shared amongst members of a population due to environmental and genetic effects. Even within Europe, faces vary among subregions and may lead to confounding in genetic association studies if unaccounted for. Genetic studies use genetic principal components (PCs) to describe facial ancestry to circumvent this issue. Yet the phenotypic effect of these genetic PCs on the face has yet to be described, and phenotype-based alternatives compared. In anthropological studies, consensus faces are utilized as they depict a phenotypic, not genetic, ancestry effect. In this study, we explored the effects of regional differences on facial ancestry in 744 Europeans using genetic and anthropological approaches. Both showed similar ancestry effects between subgroups, localized mainly to the forehead, nose, and chin. Consensus faces explained the variation seen in only the first three genetic PCs, differing more in magnitude than shape change. Here we show only minor differences between the two methods and discuss a combined approach as a possible alternative for facial scan correction that is less cohort dependent, more replicable, non-linear, and can be made open access for use across research groups, enhancing future studies in this field.

## Introduction

Although ancestry as a descriptor continues to be a controversial topic in anthropology when describing morphological features, geographic variation is often reflected in the structure of the human face and is an important factor in facial variation studies. Historically separated populations are interbreeding more than ever before, yet geographical distance and barriers still constrain random mating and lead to genomic and phenotypic diversity^1^. These differences are predominantly evident between continents, however intra-continental variability is also quite distinct. Europe contains genetically divergent populations in close geographic regions, which has been linked to migration patterns and serial founder effects^2–4^. Specifically, Southern Europe has been described as being more genetically diverse than other European regions, due to influences from both the Middle East and Africa^2,5^. On the other hand, Northern Europe has less diversification as these individuals stemmed from a smaller population of founders^3,4^. These genetic differences, alongside climate-related adaptation and selection, are reflected in the shape of the modern human face^5–7^. Previous publications have analyzed facial differences between individuals from a handful of European countries and discovered variation predominantly visible in the nose, brow, and lips area^8–10^, but a systematic comparison across broad geographic regions within Europe is missing.

Over the last decade, more than 25 genome-wide association studies (GWAS) have been performed that have mapped genes for facial traits in numerous populations^11^. To avoid spurious associations due to ancestry signals, facial GWAS studies frequently reduce cohorts to specific populations (most often Europeans) and correct both genomic and phenotypic data for genetic ancestry. Typically, these studies utilize the first four genetic ancestry principal components (PCs) to correct for European genetic ancestry. However, there is little data on precisely how these genetic PCs are describing and correcting for ancestry within the face. Furthermore, this methodology is highly debated in current literature, with Principal Component Analysis (PCA) being criticized to be highly sensitive to outliers when the data is normalized, which often leads to misalignment such as shrinkage (when new data gravitates to the middle)^12,13^. In addition, using genetics to describe facial ancestry (ancestry effects on facial variation) assumes that phenotypic variation can be adequately accounted for by genetic variation alone. This, however, does not take into account that facial variation may be larger than the genetic variation due to phenomena such as epistasis and other post-translational factors^14^. Only if these modifications correlate to regional genetic variation will they be incorporated, while significant phenotypic variation correlated with nonsignificant genetic PCs or no genetic correlation will be missed.

Yet ancestry must be accounted for, as even within Europe population stratification is evident, showing clear genetic and phenotypic ancestry signals within the continental population^15^. While genetic studies prefer to use genetic methods for describing ancestry, anthropological studies rely more heavily on phenotypic variation. In these studies, input data is often separated into ancestral groups and analyzed within these groups, and population differences are described by comparing the geographic mean faces, also known as consensus faces^8,9^. These consensus faces reflect the facial features common to a population due to their shared genetic and environmental influences, as well as selective pressures, and as such, their shared ancestry^14^. As they are not derived from genetic components, these faces describe phenotypic differences that may also account for gene–gene interactions and post-translational modifications. These generalized ancestry depictions focus more on broader ancestry signals, whereas genetic PCs describe an individual’s ancestral position within a space specific to the reference populations used. Although in anthropology the groups are often generalized geographic groups, there remains the possibility to combine both the genetic and anthropological approach; by defining sub-populations based on genetics but describing ancestry within these populations using consensus faces.

In this report we explore facial ancestry within Europe inferred from both genetic facial ancestry (derived from visualizing genetic PC score effects on the face) and phenotypic facial ancestry (consensus faces), using geometric morphometrics of more than 7000 landmarks. A comparison of the two methods illustrates that genetics do not always match phenotypic separation, yet overall, the differences are minor. We discuss the benefits and disadvantages of both approaches including the possibility of combining both as an alternative method for understanding and correcting for ancestry in future genetic studies.

## Materials and Methods

**Figure 1:**
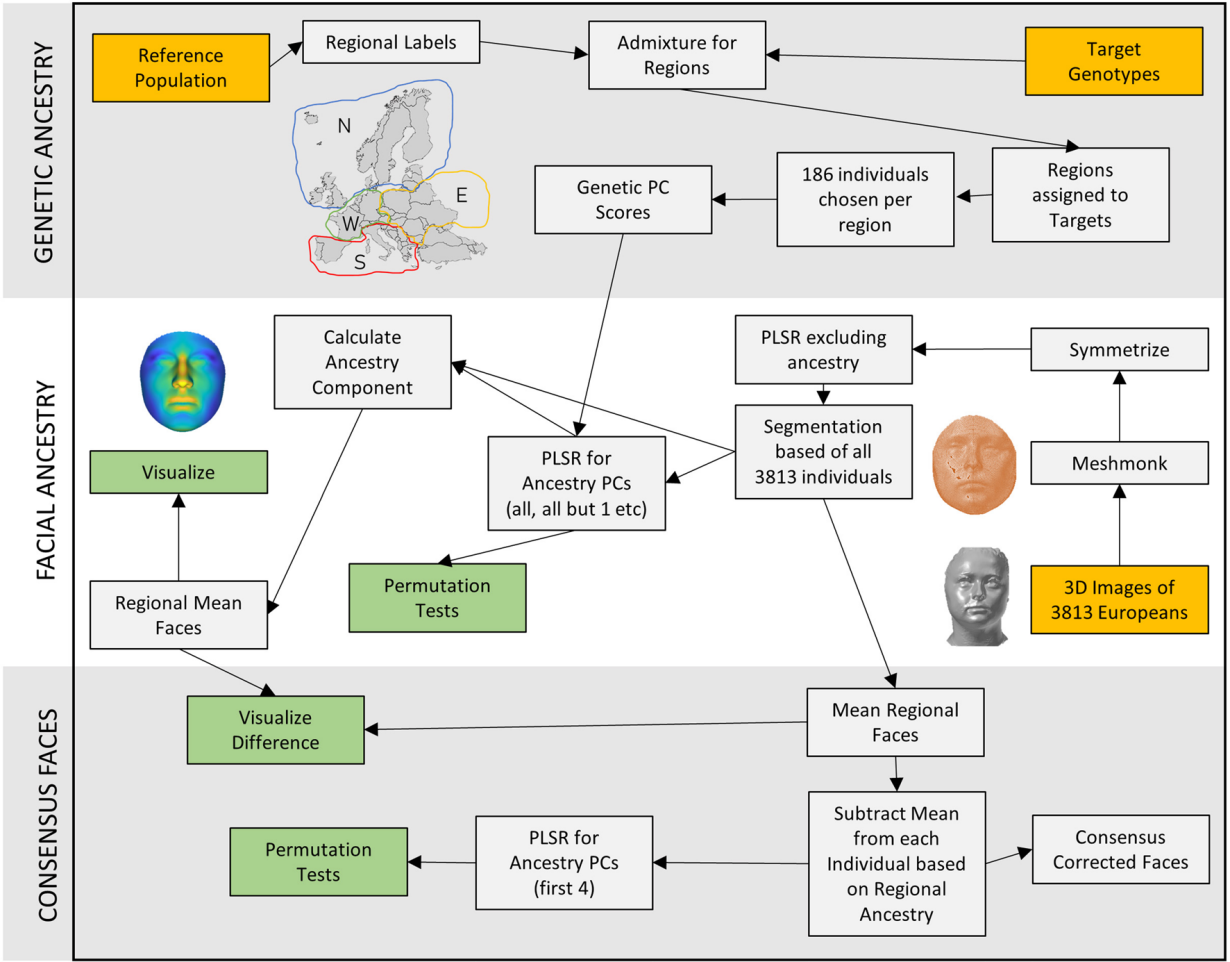
Visual overview of the computational approach to facial landmarking and association testing that was utilized in this study. Yellow indicates input data, green indicates output.

A systematic overview of the methods described below can be found in Fig. 1.

### Participant recruitment and genotypic data

The samples used for analysis were from the United States dataset of White et al.^16^. These samples were compiled from three independent datasets: Indiana University-Purdue University Indianapolis (IUPUI),Indianapolis, IN, Pennsylvania State University (PSU), University Park, PA, and the 3D Facial Norms cohort (3DFN)^17^. Each study site obtained approval from an institutional review board. 3DFN dataset: Pittsburgh, PA (University of Pittsburg Human Research Protection Office IRB00000319|PRO09060553 and RB0405013); Seattle Children’s Hospital, Seattle, WA (Seattle Children’s Institutional Review Board (IRB) IRB00000277 and IRB00009311|IRB 12107); University of Texas, Houston, TX (UT Health Committee for the Protection of Human Subjects|HSC-DB-09-0508); and University of Iowa, Iowa City, IA (University of Iowa Human Subjects Office IRB00000100|IRB 200912764 and 200710721). Pennsylvania State University dataset (Pennsylvania State University—The Human Research Protection Program IRB00000046 & IRB00000047|IRB 13103, IRB 45727, IRB 2503, IRB 44929, 4320, IRB 44929, IRB 1278) collected at various locations: Urbana-Champaign, IL; New York, NY; Cincinnati, OH; Twinsburg, OH; State College, PA; Austin, TX; and San Antonio, TX, and University of Cincinnati, Cincinnati, OH (University of Cincinnati—Human Research protection Program IRB00000180|IRB 2015-3073);. IUPUI dataset (Indiana University Institutional Review Board (IRB) IRB00000222|IRB-IUB 1409306349): collected in Indianapolis, IN and Twinsburg, OH. The participants of each study provided written informed consent before participation. Individual’s identity was protected by storing data on secured servers and by assigning anonymous identification numbers. Raw data could only be accessed by those with previous ethical approval. All experiments were performed in accordance with relevant IRB guidelines and regulations. Our subset (n = 3814) for analysis excluded any samples that were missing genetic or image data, under the age of 18, or known to have ancestral backgrounds with historical deviations from assortative mating.

Information on the genotypic data used by the independent participant sample datasets can be found in the supplementary methods of^16^. We compiled our subset of non-imputed participant data based on the existing overlap of autosomal single nucleotide polymorphisms (SNPs) (n = 132,001). We combined a reference data panel (described below) with our participant data, and this further reduced the total number of shared markers (n = 58,674). Complete genetic data overlap of our participant and reference data promotes consistent ancestry estimation parameters for downstream supervised ADMIXTURE analysis.

### Population reference data

A European reference dataset (n = 1166) was compiled of samples with varying geographical origins within Europe to represent the North (n = 414), South (n = 438), East (n = 234), and West (n = 80) cartesian regions according to definitions of the four regions by the United Nations (UN) geoscheme (found at https://unstats.un.org/unsd/methodology/m49/). These samples were selected from the Human Genome Diversity Project^18^, 1000 Genomes Project^19,20^, Balto-Slavic speaking populations^21^, George Busby’s genotype data for a set of 163 worldwide populations^22^, a genome-wide study of the Jewish population^23^, Genetics from Turkish-speaking Nomads^24^, Siberian Genome^25^, the Genetic Atlas of Human Admixture^2^, individuals from the Caucasus^26^, and the Euro180 cohort from^27^. A detailed description of the samples used can be found in Supplementary Tab. 1 online.

### Regional assignments

We assigned the UN regional label to each reference sample based on the country label provided by their respective publication’s demographic data. Using the genetic reference set and these regional identifiers, we performed a supervised analysis in ADMIXTURE^28^ to calculate the North, South, East, and West ancestry proportions for our participant data.

**Figure 2:**
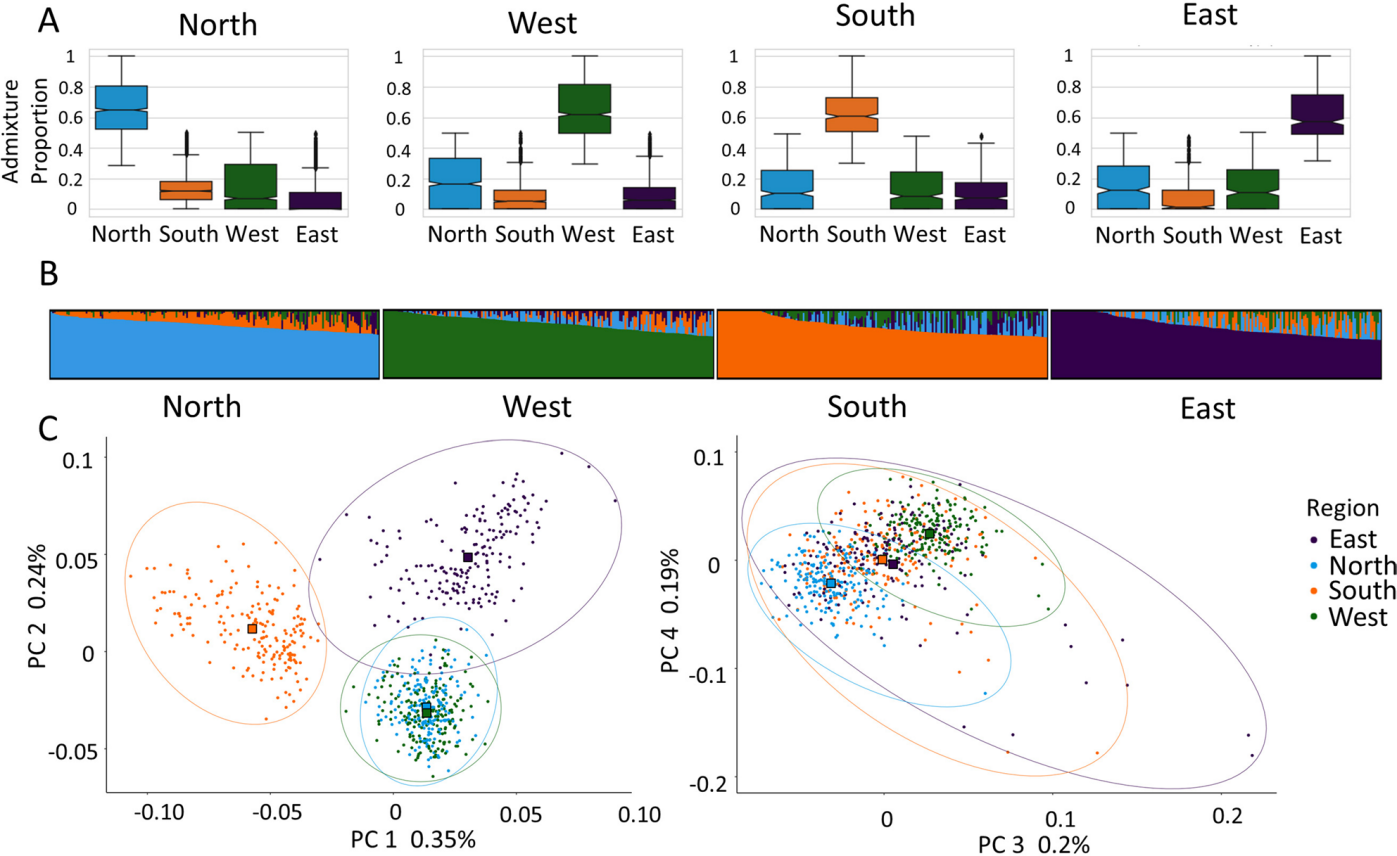
Regional assignment analysis for the participant data, (**A**) the distribution of admixture proportions for all samples assigned to the specific region based on their maximum ancestry proportion (n = 3814), (**B**) supervised ancestry proportions for the randomly selected participants (n = 186 per region) in each region above the median (observed in (**A**)) where each bar is an individual’s ancestry proportions depicted by the color that total 100% and plotted with CLUMPAK^52^, (**C**) genetic principal components 1–4 of the four regions (n = 744), including an ellipse^53^ estimated with the Khachiyan algorithm to depict the enclosed cluster. The square shapes represent the mean combination of both PCs for each region.

Each of the participants was assigned a regional label that corresponded with their maximum contributing ancestry proportion. The regional groups were then subjected to a minimum required proportion threshold that included individuals whose main ancestry proportion was greater than or equal to the regional group’s main proportion’s median value (Fig. 2A, B). The supervised ancestry proportion estimations were used solely for the selection of participants that would fully represent each region due to their similar quantitative genetic ancestry. 186 individuals from each region were randomly chosen to provide an even distribution of samples across regions, excluding the East where all participants were used. In total, there were 744 individuals for the following 3D facial morphology analyses. An overview of countries, medians, and regional cohorts can be found in Table 1.

**Table 1:** Genetic ancestry distribution of the four cartesian regions of Europe and composition of the regional cohorts.

Region	Countries in reference population	Supervised admixture: participants with maximum admixture per region	Full range of targets with a max admixture	Supervised admixture: participants above the regional median	Full range median	Randomized subset of 186 range (greater than or equal to Median)	Number of females	Number of males
North	Latvia, Estonia, England, Finland, Ireland, Lithuania, Norway, Scotland, Wales	1660	0.282–1	830	0.648	0.649–1	93	93
South	Greece, Italy, Sicily, Spain, Portugal	460	0.299–1	230	0.605	0.697–1	102	84
East	Russia, Slovenia, Slovakia, Belarus, Bulgaria, Hungary, Poland, Romania, Ukraine	374	0.315–1	186	0.571	0.571–1	124	62
West	Germany, Austria, France	1320	0.294–1	660	0.619	0.619–1	93	93

An individual level PCA was performed on the target samples (n = 744) with genotypes from the shared markers (n = 58,674) using PLINK2 '--pca' flag and visualized using the assigned regional labels (Fig. 2C)^29,30^. These genetic PCs were used in downstream genetic ancestry correction as described below and all further mention of genetic PCs refer to those describing genetic ancestry variation.

### Facial scan preparation and correction

The 3D images were registered using the MeshMonk registration framework^31^ in MATLAB (R2021b). An anthropometric mask of 7160 spatially dense quasi-landmarks was aligned and morphed to the shape of each facial scan. An initial, scaled, non-rigid alignment was performed using 5 manually placed landmarks (pronasale, endocanthion, chelion), a second, non-rigid, iterative registration, where neighboring points move together based on symmetrical correspondence and weighted k-neighbors, was performed to morph the mask onto each individual’s face. These images were symmetrized by averaging the original and its reflection (inverted X coordinate) and aligned in space via Procrustes superimposition with scaling, as only shape was relevant for this study (centroid sizes showed no significant difference between groups (ANOVA p = 0.5)).

The 744 symmetric facial scans were corrected for covariates by regressing the 744 individuals × 7160 landmarks matrix of Procrustes-aligned and scaled landmark coordinates onto sex, age, age-squared (to remove residual age effects not accounted for by a linear age covariate), height, weight, facial (centroid) size, and camera system (2 systems coded as -1 and 1) using Partial Least Squares Regression (PLSR). The corrected faces were then generated by adding the coordinates of the mean shapes back onto the residuals of the regression. Genetic PCs were not corrected at this stage.

### Permutation testing and facial visualization of genetic pc effects

Permutation tests based on residuals from the PLSR, with 10,000 permutations of the model were performed to determine if correction for ancestry for the first four genetic PCs via PLSR significantly altered the shape of the facial scans and thus described a significant amount of the facial variation present. For computational efficiency, shape variation was reduced by PCA of the landmark coordinates and only those explaining up to 96% of variation were retained (depending on the regional group; between 15 and 20 PCs). The scores on each PC were normalized to have unit variance. Genetic PCs were tested individually and in combination. Both the p-value and percentage of variance explained (total R^2^ value) were calculated. This test was run with varying input groups: n = 744 (all sub-regions), n = 186 (per sub-region), or n = 372 (between two sub-regions) to determine which geographical gradients each PC described.

The 744 facial scans, corrected previously for all covariates excluding ancestry, were then corrected for genetic ancestry via PLSR using the first four ancestry PCs. The difference in landmark location before and after ancestry correction was deemed the genetic facial ancestry effect. This effect was multiplied by four (for better visualization) and added onto an average of all 744 faces corrected for all covariates: including the first 20 genetic PCs. The difference in the resulting faces between ancestry correction via PLSR using all four PCs and using only three PC (one excluded) was calculated to represent the effect of the excluded PC and exaggerated eight times for visualization purposes only. For regional PC faces, the difference between ancestry-corrected faces (first four PCs) and the faces before ancestry correction was calculated, averaged per region (n = 186), and added onto the average face corrected for all covariates. Effects were exaggerated four times for visualization.

The maximum ancestry shift was determined by calculating the absolute distance between the pre- and post-ancestry correction facial scans per landmark per individual. The maximum distance over all individuals per landmark was used. Facial ancestry effects were visualized as normal displacement maps (magnitude of shape change), and angle differences (direction of shape change).

### Regional consensus faces

Consensus faces were created by averaging the 186 faces per region, after correction for all covariates but excluding ancestry, and subsequent Procrustes alignment. Each of the 744 individuals was then corrected for ancestry by subtracting their region’s consensus face from their individual facial scan. The resulting residual was added to the average face from the initial PLSR (the same average face as added to the residuals after the ancestry PLSR correction) to provide us with each individual’s consensus corrected face. A permutation test for the ancestry PCs was performed after consensus face correction to analyze if the genetic PCs still described a significant amount of variation after consensus correction.

## Results

### Determination of subpopulations

Sample size distribution for the target data (n = 3814) showed greater representation above the regional median from the North (n = 830) and West (n = 660) regions and the least representation from the East (n = 186) and South (n = 230) regions. Within the regional subsets (n = 186), male and female distribution was equally balanced for the North and West regions (males = 93; females = 93), while the East (males = 62; females = 124) and South (males = 84; females = 102) regions were limited in the number of male individuals available above the regional median. Genetic PC1 separated out the South population while PC2 created the gradient East-South-Northwest. PC3 differentiated North from West while PC4 explained some additional variation (Fig. 2C).

### Effects of ancestry correction using genetic PCs

**Figure 3:**
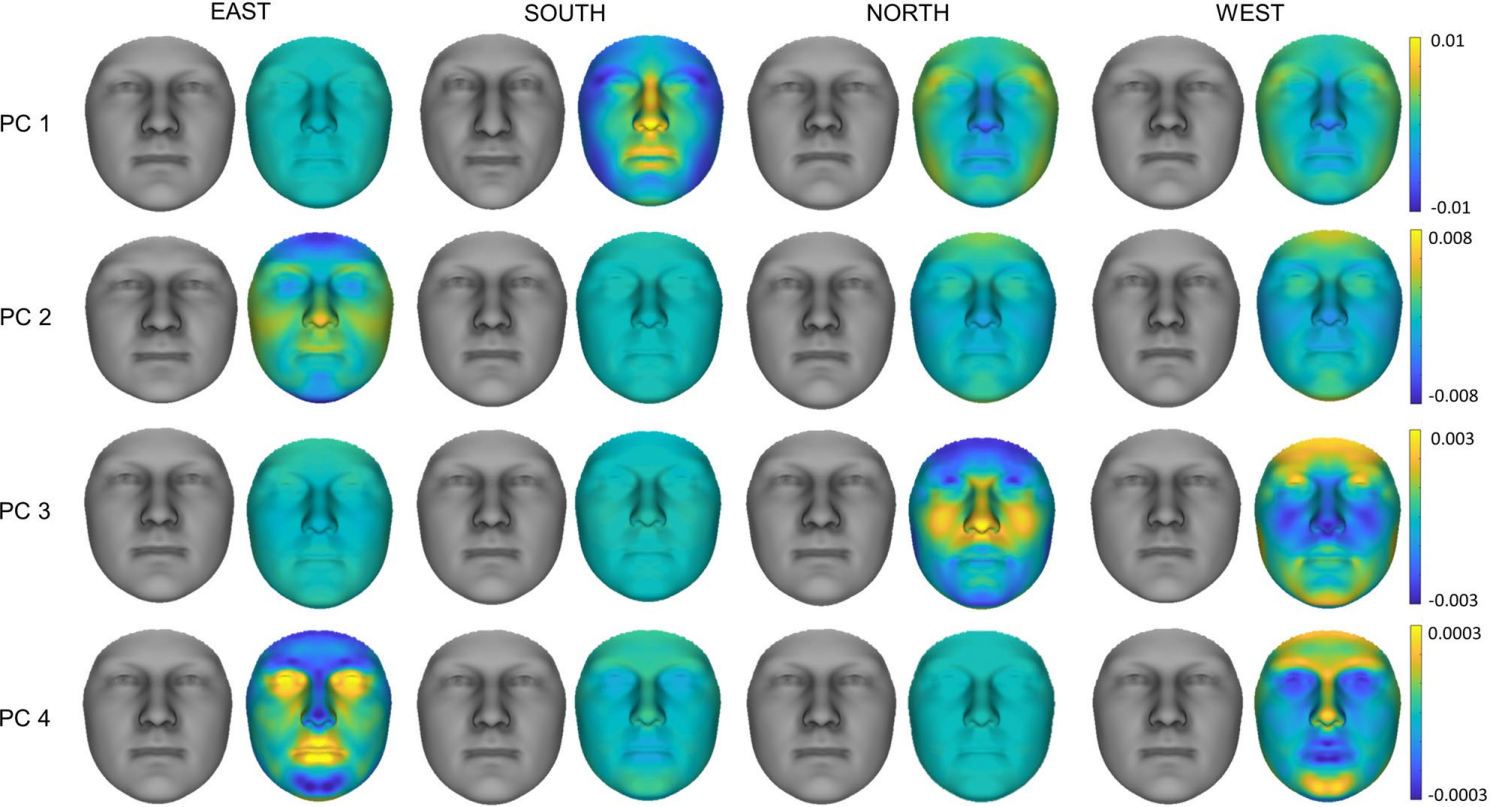
Visual effects of the first four genetic PCs on the facial phenotype representing the four regional populations, North, South, East, and West. Exaggerated faces (8X; shown in grey) and normal displacement maps (magnitude of inward/outward movement) of PC effects on regional faces (n = 186) are shown. Displacement maps are normalized to the same axis per PC for comparison between regions.

Genetic PC1 shows a protrusion of the nose bridge, nose tip, and lips in the Southern face, with an opposite effect of a slightly wider face and flatter mid-face in the North and West. Little to no effect is seen in the East (Fig. 3). Its effect displays a majority S vs N/W gradient phenotypically, where the South shows the largest effect, the East shows no effect, and the North and West show an effect in the opposite direction (Fig. 3). Yet, an S vs N/W vs E gradient is evident in the genetic PC plot (Fig. 2C) indicating a slight discordance between phenotype and genotype. PC1 significantly alters the shape in both the South-West and South-North comparisons, as well as within the Southern population (Table 2). This implies that PC1 also describes intra-region variation within the South. PC2 displays a wider and shorter face in the East as well as a protrusion of the nose tip. Slight opposite effects are seen in the North and West (Fig. 3). An E vs W gradient with some effect in the North is visible in both the genetic and phenotypic effects. Little to no effect is seen in the South (Fig. 3). South-East and East–West gradients are affected by PC2, as well as all regions combined. No significance is seen within a region (Table 2) suggesting PC2 does not describe any intra-region variation. Neither PC1 nor PC2 show variation between the North and West. PC3 appears to explain opposing effects in the North and West, with a more prominent nose and cheeks in the North and a more prominent chin and brow in the West (Fig. 3). Little to no effect is seen in the South and East. Genomic PC3 scores show a separation of West and North (Fig. 2C), however, this PC shows no significant effect on the facial phenotype (Table 2). PC4 appears to exhibit the smallest effect with a less prominent forehead, nose, and chin in the East and an opposite effect in the West while genetic plots show a North-West separation only. PC4 only showed significance within the West population but not between regions (Table 2). As only PC1 and PC2 were significant within the entire cohort, all regions can be separated apart from North vs West, which show negligible differences. In total, the first 4 PCs describe 1.6% of the facial variation within our cohort.

**Table 2:** Significance of genetic PC correction on facial phenotype.

PC	ALL	North–East	South–East	East–West	North–South	North-West	South–West	North	South	East	West
1	**0**	0.625	**0.023**	**0.023**	**0**	**0.015**	**0**	0.293	**0.001**	0.176	0.205
2	**0.003**	0.725	**0.036**	**0.013**	0.948	0.701	0.612	0.998	0.415	0.182	0.089
3	0.204	0.485	0.554	0.544	0.374	0.49	0.599	0.349	0.667	0.553	0.111
4	0.444	0.215	0.43	0.163	0.074	0.293	0.393	0.263	0.552	0.416	**0.02**
5	0.56	0.456	0.428	0.198	0.34	**0.047**	**0.048**	0.247	0.157	0.324	0.326
6	0.488	0.199	0.592	0.622	0.05	0.4	0.202	0.095	0.08	0.413	0.518
7	0.64	0.62	**0.03**	0.515	0.155	0.114	0.589	0.098	**0.014**	0.186	0.565
8	0.409	0.314	0.087	0.545	0.878	0.918	0.94	0.912	0.689	0.69	0.951
9	0.064	0.162	**0.04**	0.104	**0.025**	0.749	0.184	0.438	0.053	**0.037**	0.386
10	0.838	0.379	0.388	0.276	0.19	0.937	0.412	0.558	0.242	0.859	**0.027**
11	0.234	0.408	0.085	0.55	0.265	0.524	0.068	0.499	**0.038**	0.438	0.52
12	0.453	0.402	0.113	0.208	0.176	0.228	0.097	0.414	0.107	0.116	**0.011**
13	0.128	0.304	0.374	0.299	0.511	0.052	0.088	0.234	0.832	0.167	0.061
14	0.412	0.628	0.162	0.369	0.726	0.766	0.304	0.512	0.441	0.058	0.806
15	0.489	0.133	**0.035**	0.281	0.929	0.823	0.728	0.906	0.22	0.062	0.258
16	0.1	0.599	0.25	**0.046**	0.38	0.538	0.17	0.594	0.615	0.27	0.134
17	0.32	0.393	0.27	0.112	0.183	0.279	0.351	0.351	0.181	0.073	**0.011**
18	**0.001**	0.068	**0.023**	**0.009**	0.295	0.321	**0.017**	0.547	0.206	**0.002**	0.064
19	0.892	0.916	0.953	0.39	0.367	0.874	0.802	0.928	0.346	0.08	0.958
20	0.882	0.872	0.986	0.515	0.766	0.658	0.721	0.575	0.412	0.964	0.77

The table shows p-values from permutation (10,000 iterations) testing on segment 1 (entire face) for different input groups. Significant results (p-value < 0.05) are highlighted in bold.

A permutation test was also performed on genetic PCs 5-20 to elucidate whether additional PCs may also describe phenotypic ancestry. PC18 was found to be significant in our population in multiple regional comparisons as well as over the entire cohort (0.2% facial variation explained). It was also significant in the East (0.9% facial variation explained) and showed the highest pairwise significance in the East–West gradient. Its high significance in mostly Eastern comparisons may indicate some Eastern-specific variation that does not exist between regions. No other PCs showed overall significance, but some (PC5,7,9,15,16) showed significance in one or more between-region comparisons and these remain significant even after ancestry is removed (see Supplementary Tab. S2 & S3 online). In total, PC1-20 described 3.78% of the genetic variation and 3.27% of facial variation.

### Combined genetic ancestry effects and comparisons per region

**Figure 4:**
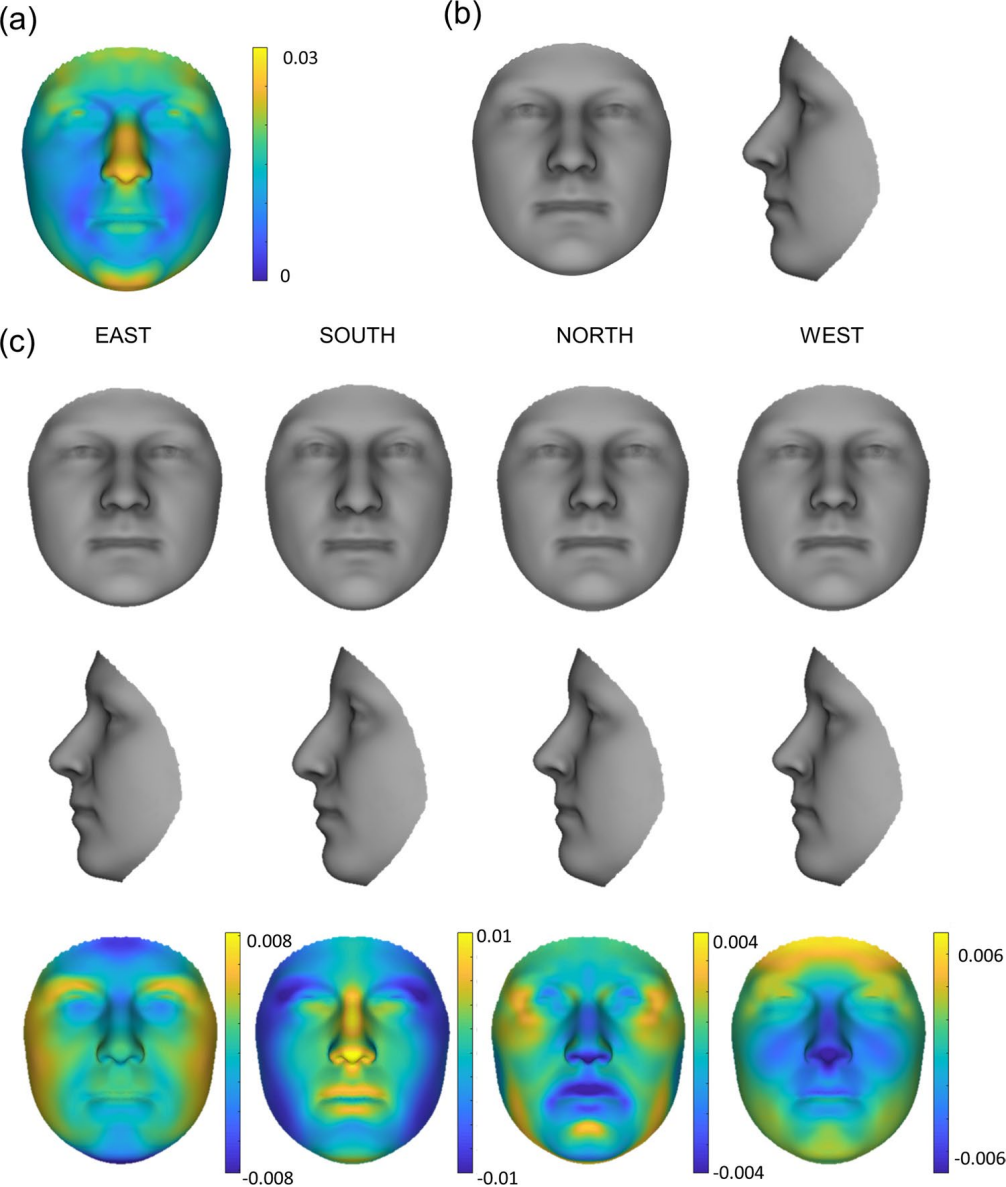
Regional ancestry effect: (**a**) maximum ancestry shift per landmark, (**b**) average face with ancestry removed for comparison, (**c**) average regional ancestry effect multiplied (4X) and normal displacement maps (magnitude of inward/outward movement) of ancestry effect (n = 186 per region).

When combining the effects of the first four genetic PCs, the nose demonstrated the greatest maximum ancestry shift alongside the chin. The smallest shift was seen in the cheeks and nasolabial area (Fig. 4a). By combining the effects of genetic PC1-4 (1% facial variation explained) and exploring facial ancestry on a regional level, the South exhibited the largest genetic facial ancestry effect, followed by the East, the West, then the North. The South showed a down-turned nose tip, a more convex nose bridge, and in general more prominent mid-face. The Southern face is slimmer and the jaw narrower. A slight protrusion of the mid-face is seen in the East, alongside a shorter and wider overall face. In contrast, the West is characterized by an inward rotation of the face leading to a protrusion of the forehead and chin and inward movement of the mid-face. Although minimal, the North shows a wider jaw, slight protrusion of the outer corners of the eye socket, a slightly upturned nose tip, and flatter lips in comparison to the corrected average (Fig. 4b and c).

**Figure 5:**
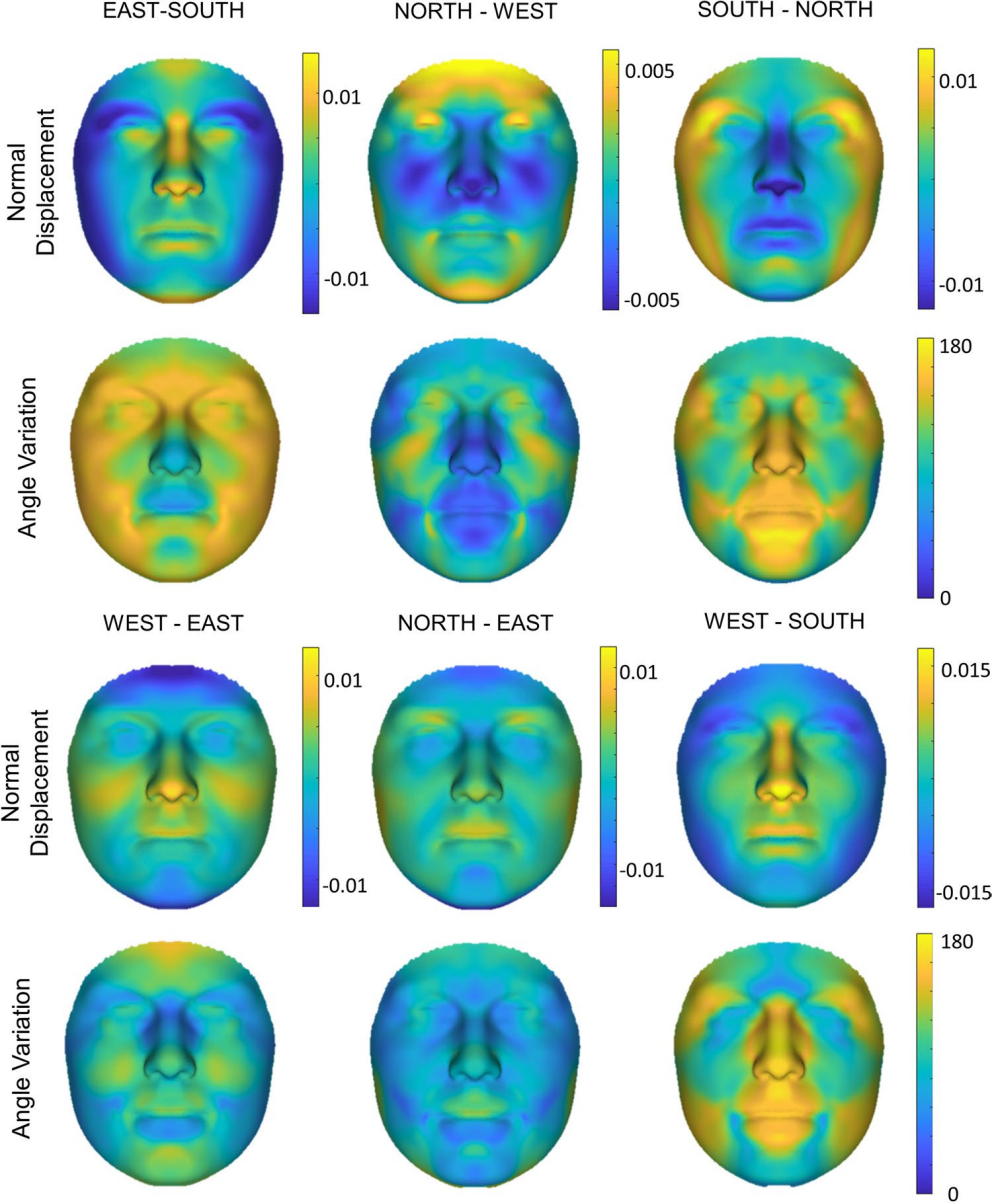
Regional ancestry comparisons. Normal displacement (magnitude of inward/outward movement) and angle differences (directional differences in shape change in degrees) between the average regional faces (n = 186 per region).

When regions were compared to each other, the East and South show similar shape changes in the nose tip and upper lip with the South having a stronger effect. Opposite effects are visible in the width of the face and shape of the eyes. A flatter mid-face is seen in both the North and West. The South to North gradient is defined by a slimming of the face and protrusion with a downward turn of the nose and mid-face. Differential effects were also visible along the West to East gradient in the cheeks, chin, and forehead. Comparison of the West to South showed the largest difference with opposing effects in the midface, jaw, and outer eye area. North to East showed very little shape differences but larger magnitude differences (Fig. 5).

### Phenotypic consensus face comparison

**Figure 6:**
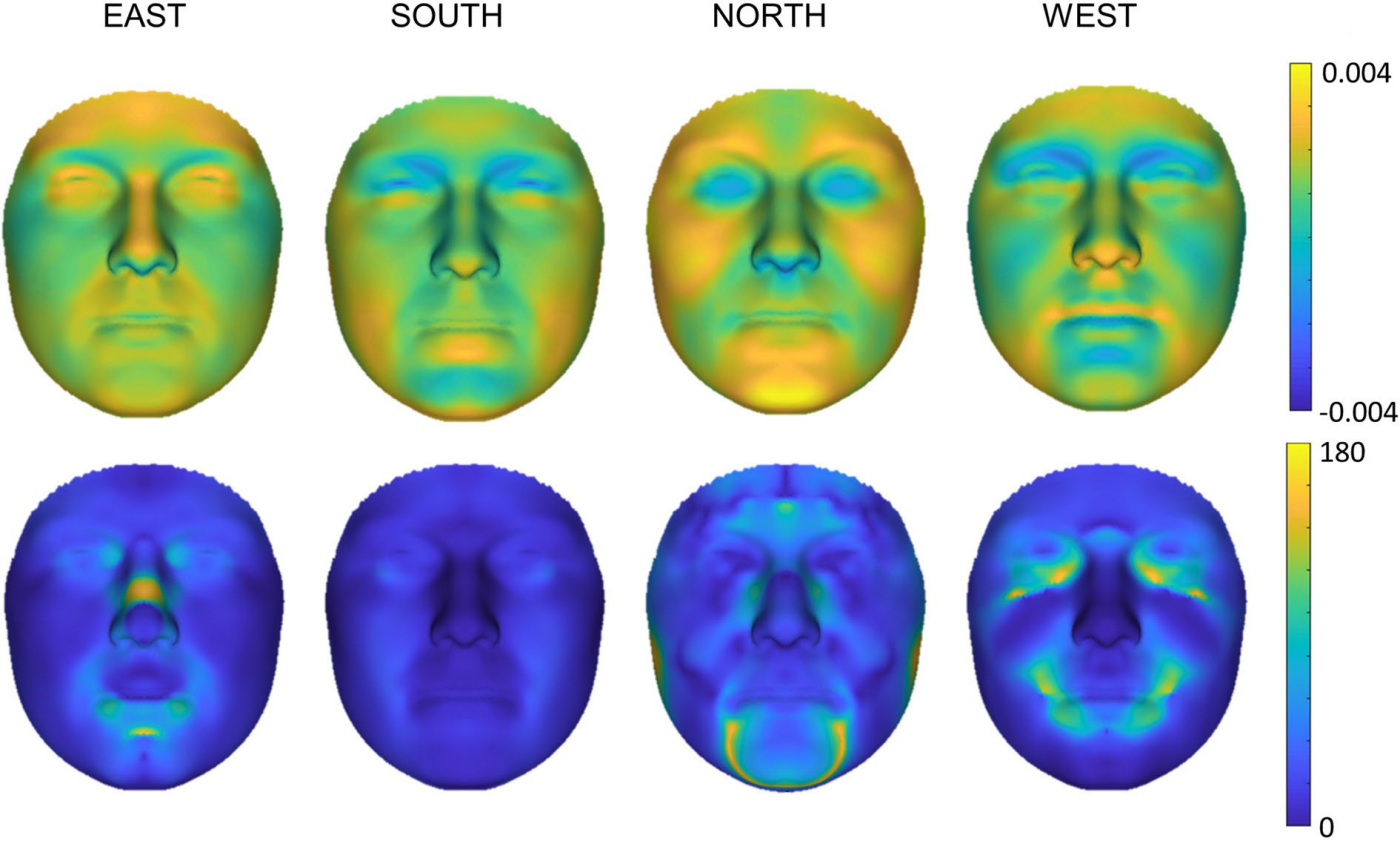
Consensus and genetic PC face comparisons. Normal displacement (top row—magnitude of inward/outward movement where yellow shows higher magnitude in consensus faces and blue in PC faces) and angle differences (bottom row—directional differences in shape change in degrees) between consensus faces and average genetic PC faces per region (n = 186 per region).

A direct comparison of the derived consensus faces to the regional faces created from the genetic PCs showed only minor angle (shape direction) differences, most visible in the Eastern nose. The eyes, cheeks, and upper chin showed the least magnitude (normal displacement) differences in all but the East. The consensus faces showed wider cheeks and forehead in the North, with a larger jaw, and displayed the largest magnitude changes over all regions. For the West, a wider jaw, taller forehead, and increased protrusion of the nose tip and upper lip were visible in the consensus face in comparison to the genetic PC face. The least difference in magnitude was visible in the South with a wider jaw, longer chin, and protruding lower lip in the consensus face (Fig. 6). Visually there is little to no difference between the regional averages (see Supplementary Fig. S1 online).

Permutation testing using genetic PCs after consensus correction showed that genetic PCs 1 and 2 were no longer significant. PC3 showed an increase in p-value from 0.2 to 0.99, indicating it was defined within the consensus face and does describe facial ancestry (albeit not significantly). PC4 and PCs 5-20 remained unchanged, suggesting these PCs do not describe phenotypic ancestry between regions within this cohort (see Supplementary Tab. S2 online).

## Discussion

Although facial ancestry has been explored previously within Europe, it has frequently been limited to only a few populations and has less dense landmarking techniques. In addition, there is a paucity of research to link the genetic PCs (which are used to correct ancestry) to facial ancestry, as well as discussions on the accuracy of using genetic scores for phenotypic correction. Here we have attempted to address these aspects by describing facial ancestry variation within Europe using two different approaches: genetic and anthropological, and visualizing the differences between the two.

One main point is evident in this research: Europe is not a homogenous population and shows ancestral facial variation. Genetic ancestry has previously been shown to mirror geography^3^, and research has shown that genetic PCs within Europe separate out the four Cartesian regions: PC1 often describes the North–South gradient, while PC2 describes East–West differences^32,33^ which correspond with our findings. Our results show that genetic PC1 defined the differences between the South and Northwest, while PC2 separated East from Northwest within our dataset. Only these two PCs significantly affected the face, as such there was no separation between the North and West phenotypically, which coincides with ideas put forward on the settlement of these regions. We also replicate previous findings on higher ancestry signals in the face within the South and East due to known migratory routes to Europe through the East and South which extended to the North and West via the serial founder effect^4^. As such, the genetic variation observed in the Northwest was much smaller as it stemmed from fewer founder individuals, in addition to gene flow between these regions which may in part account for similarities seen within our data^4^. Furthermore, these regions migrated back to the Southeast during the last glacial maximum, reintegrating their genetics into the Southeast and increasing variation there. Post-glacial recolonization included even fewer individuals, reducing genetic variation in the North once again^4,34^. Eastern Europe is closest to Africa and Asia, thus, migration from these regions brought new genetics to Eastern Europe first, increasing variation in a gradient from East to West and South to North^2,4^. In addition, specific phenotypes are highly affected by ancestry due to climate adaptation^6^. For example, our data show a larger, more downturned nose in the South, with a smaller more upturned nose in the North. Noses in warmer regions display a larger, wider, and more downturned nose, allowing the humid air to be cooled; in colder regions, a narrow high nose allows the air to be moistened and warmed^7,35,36^. Moreover, variations in chin shape found within our data set may also suggest a link to migration patterns of hunters/gatherers and differences in diet^37^. Furthermore, we replicated European facial data; when considering an Italian (South), Lithuanian (North), and German (West) population, previous research has shown that the North and West had a rounded nose tip, while the South showed a straighter nose tip, high eyebrows and slightly upturned mouth corners^10^.

Although these facial differences between geographic regions have been linked to N–S and E–W gradients described by the first 4 genetic PCs, a slight discourse was visible between the genetic data and its phenotypic expression within our dataset. Both genetic plots and facial ancestry describe the same gradient for genetic PC2, whereas for PC1, East showed an opposing position to South genetically, but an intermediate phenotype within the face indicating that phenotypic variation may not be entirely represented by genetic differences. PC18 describes a significant amount of facial variation, but not genetic variation. The facial variation described by PC18 is thus missed with only a genetic depiction of ancestry and may be linked to post-translational modifications or gene–gene interactions not accounted for or correlated to significant genetic PCs. However, it was not influenced by the consensus faces and thus may describe intra-region variation and not variation between our geographic regions. Yet overall, consensus faces and genetic PC faces showed similar phenotypes. Facial variation described by genetic PC1 and PC2 was completely accounted for by the consensus faces and did not describe any further variation after the faces were adjusted for the consensus faces. Although genetic PC3 was not significantly associated with facial shape in our smaller cohort, when testing a larger population of European individuals (n = 3814), it did pass the significance threshold (data not shown). Importantly, facial ancestry variation from PC3 was completely described by the consensus faces, indicating that it does seem to describe facial ancestry. On the other hand, genetic PC4 described no significant amount of facial variation. Neither PC4 nor PCs 5-20 facial variation was explained by the consensus faces suggesting that these PCs describe variation that may not be linked to regional ancestry, though possibly ancestry on a subpopulation level. It is important to note, however, that correcting for insignificant PCs does not significantly change the face shape and correcting for insignificant covariates has been shown to not negatively affect results, although it does reduce the degrees of freedom^38^. Overall, both approaches showed similar facial ancestry shape changes, with slight differences in the magnitude of the shape change.

Although both genetic PC facial correction and phenotypic consensus face correction describe comparable facial ancestry signals, each has its benefits and drawbacks. For ancestry analyses, ancestral populations must first be determined and described. The main benefit of PCs are that they reduce the dimensionality of genetic data down to only a few summary variables which is easily visualized on plots that seem to separate ancestral populations. Individuals are projected onto a space along these PC axes, and distances between clusters are assumed to reflect the genetic, geographic, and phenotypic distances between populations. Yet statistically these only describe the greatest variation which may or may not be entirely linked to ancestry^12^. Genetic ancestry in itself is arguably a non-linear construct that we have long forced into a PCA space, which has seen a surgency of alternative methods to better facilitate its reduction^12,39–41^. Typical anthropological practices of describing ancestral variation using consensus faces often rely on available self-reported ancestry, although recent work is moving towards the inclusion of genetic ancestry^42^. However, in our modified consensus face approach, this is overcome by using genetics as a prior through an unbiased classification of the region in which an individual belongs via ADMIXTURE. The benefit over PCA is that it is based on contributions from each of the source populations (k) and less so on their individualized placement in an ancestry space. Although choice of source population groups certainly influences categorization of individuals, it seems a more conservative approach overall.

Once genetic ancestry or ancestral groups are described, their relationship to the phenotype must be modeled. Linear regressions further limit the amount of shape changes that can be described in the face and are not efficient at considering covariate interactions when given multiple variables for ancestry. Nonlinear regressions and mixed effect models are a solution but can quickly become highly complex. Although consensus faces model a nonlinear relationship between ancestry and phenotype, they require a sufficient number of 3D facial scans to adequately generate the consensus face of the region, which can be a difficult task as one must ensure that all clusters within the research cohort are adequately represented for phenotypic ancestry correction. Yet the main advantage is that consensus faces describe the entirety of phenotypic variation and are not reduced to a few linearized genetic gradients between populations.

A distinct advantage of the consensus correction approach relates to reproducibility and comparability between research studies. With PC scores and linear regressions, these can vary depending on which individuals are included, leading to differing results for a phenotype that should not vary if new individuals are added as facial ancestry is fixed. While standardizing the choice of reference populations may help ensure more comparable PC scores, the regression models will still fluctuate. On the other hand, once a consensus face is created to represent a particular region, it can be shared across research groups without ethical implications as it does not describe an individual. As such, the consensus face bypasses issues with cohort-specific correction effects and varying methodologies for covariate correction and remains a less complex, and less error-prone, method of describing facial ancestry variation.

In exploring these two ancestry facial correction approaches, it is important to also describe the limitations of this study. Although we attempted to select individuals with an equal genetic variation spread, our East and South populations did show higher PC score variation than West and North. The West cluster was more compact, while a lack of East samples and a wider spread led to more diverse individuals which may have influenced the results. The lack of individuals in some regions limited our cohort to only 744. In addition, our data included only individuals that clearly clustered (K-means) to a specific region, but not every sub-population within these regions was represented. These clusters were assigned to regions using the UN geoscheme and this meant it was predetermined which countries were assigned to each region. This leaves unanswered questions as to (1) how to correct for admixed individuals, and (2) if and what are the sub-population specific effects. Our results show that PC1 remains significant within the Southern population even after consensus correction and PC18 is significant within the East, indicating that individuals vary within in their facial phenotypes within these regions, which may also be attributed to sub-population ancestry variation. Previous publications have shown that genetic ancestry can vary even within specific sub-regions^43^. Consequently, further refinement is needed with smaller subpopulations analyzed to discover which ones need individual consensus faces.

While some associations found in European facial GWAS have been replicated in other populations, many have not^44^. Especially SNPs that are fixed in global populations will not be discovered unless admixed individuals are considered^45^. From an anthropological approach, this leads to the issue that many individuals self-identify as a specific population but in reality, show varying amounts of admixture^46^. For a genetic approach, PCs are not adequate to describe admixture as it is a general model that does not consider the basics of population genetics but instead places an individual in space between two populations with the assumption that the distance to each population cluster describes its admixture proportions^35,47,48^. Nevertheless, other genetic methods such as Tractor^39^ have been described to facilitate local ancestry and admixture correction, and programs such as STRUCTURE and ADMIXTURE show positive results for predicting admixture proportions^47,48^. Potential points of discussion and future avenues of research have shown that admixed individuals usually show an intermediate phenotype between their ancestral phenotypes^49,50^, which may allow us to include admixed individuals for consensus face correction.

Although the goal of facial GWAS is to discover genes that affect individual facial variation (not ancestry), understanding how, where, and in what pattern ancestry affects the face is important not only for correction, but for exploring evolutionary events associated with phenotypic selection. It also allows us to consider the potential for improving future individualized facial predictions that require the reintegration of covariates. There have only been approximately 300 genes linked so far to human facial variation, and associated variants in/around these genes explain only a fraction of the heritability. One limiting factor is the lack of power to find more variants with low penetrance. This can stem from the relatively small sample sizes (by modern GWAS standards) available for these types of studies, or the inability to meta-analyze results for larger sample sets due to incompatible methods and the inability to share datasets. Or more recently discussed, lack of power due to simplified linear adjustment of non-linear covariates^41^.

It may be worthwhile to consider a combined approach to avoid many of the issues described above. By using ADMIXTURE to measure the proportion of genetic likelihood of belonging to a particular reference population to determine an individual’s ancestry, we do not need to rely on self-reported data. If we then apply a weighted (dependent on admixture proportions) consensus face correction, we avoid methodological issues related to PCA as well as replicability and comparability issues. This also avoids limiting cohort size by allowing the use of global populations that include admixed individuals. Although it may be difficult to collect faces for every population, previous research has shown that relatively robust facial averages can be created from approximately 30 individuals^51^. Thus, if we can create a database of consensus faces, we can increase GWAS power with larger cohorts, improved ancestry correction, and including the consideration of admixture, make the first step towards a standardized method for meta-analyses. This would also contribute to anthropology, allowing a more comprehensive characterization of facial diversity using matched genotype and phenotype sample in large cohorts across the world. Furthermore, population genetic studies would benefit from studies describing phenotypic change across regions.

## Conclusion

This study shows that ancestry cannot be ignored even within more homogenous populations. Europe demonstrates clear ancestral differences in the face, particularly within the East and South, that are similar between genetic facial ancestry and consensus faces. Thus, using consensus faces instead of genetic PCs may be a viable alternative. We propose combining both methods and describe a mixed approach to ancestry correction, using both genetic and anthropological components. By using genetic methods to define an individual’s ancestry but describe facial ancestry using consensus faces, we bypass most of the above-stated concerns. Consensus faces defined by genetic ancestry proportions are more replicable and easier to apply, simplifying both analyses and comparability of multiple studies, and may, in the future, provide more robust global GWAS and meta-analyses in craniofacial variation studies.

## Supplementary Information

Supplementary Information 1.

Supplementary Table S1.

Supplementary Table S2.

Supplementary Table S3.

## Data Availability

3D object files and genotypic data from the participants included in the PSU and IUPUI datasets were collected without broad data-sharing consent. Raw data would be an ethical and legal violation of informed consent. Raw source data for the 3DFN dataset are available through the FaceBase Consortium (https://www.facebase.org) at accession no. FB00000491.01 after institutional ethics approval and approval from the data access committee. Genotypic datasets used as references were taken from: The 1000 Genomes Project Consortium (10.1038/nature15393 & 10.1093/nar/gkz836). George Busby’s genotype data for a set of 163 worldwide populations (10.1016/j.cub.2015.08.007). Human Genome Diversity Project (10.1126/science.aay5012). Balto-Slavic speaking populations (10.1371/journal.pone.0135820). A genome-wide study of the Jewish population (10.1038/nature09103). Turkish Speaking Individuals (10.1371/journal.pgen.1005068). Siberian Genome (10.1038/nature12736). The Genetic Atlas of Human Admixture (10.1126/science.1243518). Caucasus Individuals (10.1093/molbev/msr221). The MeshMonk (v.0.0.6) spatially dense facial-mapping software is provided by KU Leuven and is free to use for academic purposes (https://github.com/TheWebMonks/meshmonk). All code used for analyses was modified from a previous publication (10.1038/s41588-020-00741-7). Further underlying code is available as part of the Matlab software (https://www.mathworks.com) as well as Plink2.0 (https://www.cog-genomics.org/plink/2.0/) and ADMIXTURE (https://dalexander.github.io/admixture/).
