# Supplementary material for: Exploring regional aspects of 3D facial variation within European individuals
Source: Sci Rep. 2023 Mar 6;13:3708. doi: 10.1038/s41598-023-30855-x (PMC9988837; doi:10.1038/s41598-023-30855-x)
Supplement: Supplementary file 1 — Supplementary Information 1. [file 41598_2023_30855_MOESM1_ESM.pdf]

SUPPLEMENTARY MATERIALS

Exploring Regional Aspects of 3D Facial Variation in a European Population

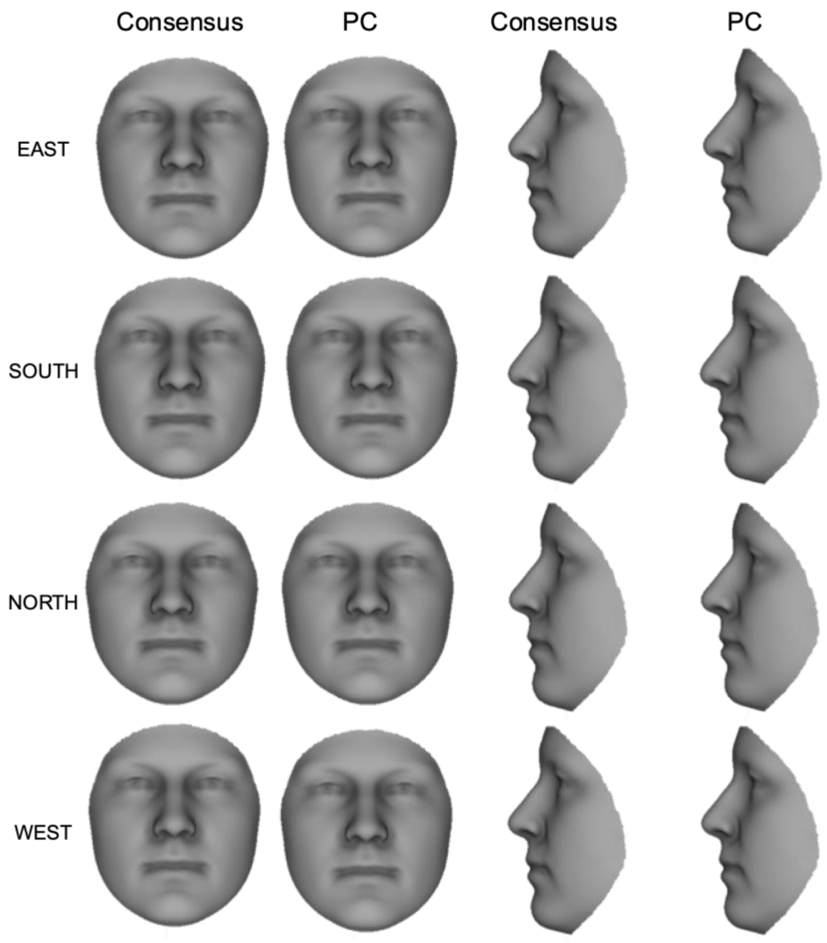

Supplementary Fig.S1: Average consensus and genetic PC regional faces.

Supplementary Tab. S1: Data sources for European reference genomes and their UN Geoscheme region assignment. These samples were selected from the Human Genome Diversity Project [18], 1000 Genomes Project [19, 20], Balto-Slavic speaking populations [21], George Busby's genotype data for a set of 163 worldwide populations [22], a genome-wide study of the Jewish population [23], Genetics from Turkish-speaking Nomads [24], Siberian Genome [25], the Genetic Atlas of Human Admixture [26], individuals from the Caucasus [27], and the Euro180 cohort from [28].

Supplementary Tab.S2: Permutation testing (10,000 iterations) for the effect of correction for each genetic PC before ancestry correction (No Corr), after consensus face correction (Pheno) or after correction for the first four ancestry PCs (Geno) on the facial shape for different regional groups (i.e. North, South, East, West comparisons). Significant values ($p<0.05$) are highlighted in bold.

Supplementary Tab.S3: Permutation testing (10,000 iterations) for the effect of correction for each genetic PC before ancestry correction (No Corr), after consensus face correction (Pheno) or after correction for the first four ancestry PCs (Geno) on the facial shape for combined regions (i.e. South + West = Southwest gradient). Significant values ($p<0.05$) are highlighted in bold.
